# Whole-Genome Sequence of *Potamopyrgus antipodarum*—A Model System for the Maintenance of Sexual Reproduction—Reveals a Recent Whole-Genome Duplication

**DOI:** 10.1093/gbe/evaf192

**Published:** 2025-11-05

**Authors:** Joseph Jalinsky, Kyle E McElroy, Joel Sharbrough, Laura Bankers, Peter D Fields, Chelsea Higgins, Cynthia Toll, Jeffrey L Boore, John M Logsdon Jr, Maurine Neiman

**Affiliations:** Department of Biology, University of Iowa, Iowa City, IA, USA; Department of Biology, University of Iowa, Iowa City, IA, USA; Department of Ecology, Evolution, and Organismal Biology, Iowa State University, Ames, IA, USA; Department of Biology, University of Iowa, Iowa City, IA, USA; Department of Biology, Colorado State University, Fort Collins, CO, USA; Department of Biology, New Mexico Institute of Mining and Technology, Socorro, NM, USA; Department of Ecology, Evolution, and Marine Biology, University of California, Santa Barbara, Santa Barbara, CA, USA; Department of Biology, University of Iowa, Iowa City, IA, USA; Department of Environmental Sciences, Zoology, University of Basel, Basel, Switzerland; Department of Biology, University of Iowa, Iowa City, IA, USA; Department of Zoology, University of British Columbia, Vancouver, Canada; Department of Biology, University of Iowa, Iowa City, IA, USA; Biorepositories Program, Mayo Clinic, Rochester, MN, USA; Phenome Ventures, Seattle, WA, USA; Department of Integrative Biology, University of California, Berkeley, CA, USA; Department of Biology, University of Iowa, Iowa City, IA, USA; Department of Biology, University of Iowa, Iowa City, IA, USA

**Keywords:** parthenogenesis, whole-genome duplication, meiosis, transposable elements, snail, Mollusca

## Abstract

Key unanswered questions in biology center on the causes, consequences, and maintenance of sexual reproduction (“sex”). Genome-driven processes are central to the evolutionary and genetic mechanisms inherent to sex, making genomic resources a fundamental part of answering these questions. We present the first genome assembly for a species that is uniquely well-suited for the study of (a)sex in nature, *Potamopyrgus antipodarum*. This New Zealand snail is unusual in featuring multiple separate transitions from obligately sexual to obligately asexual reproduction, leading to the coexistence of phenotypically similar sexual and asexual forms, a feature that is required to directly study the maintenance of sex. These separately derived asexual lineages constitute separate evolutionary experiments, providing a powerful means of characterizing how the absence of sex affects genome evolution. Our genome assembly provides critical steps toward understanding the causes and consequences of sex in this system and important resources for the rapidly growing *P. antipodarum* and molluscan genomics research community. In characterizing this genome, we uncovered unexpected evidence for a recent whole-genome duplication (WGD) in *P. antipodarum*. This discovery sets the stage for using *P. antipodarum* to evaluate processes of rediploidization following WGD and to assess whether WGD might drive transitions to asexuality.

Significance Why sexual reproduction (sex) is so common remains unclear in light of its profound costs relative to asexual reproduction. Understanding the predominance of sex remains a major evolutionary puzzle, hindering progress in fields ranging from cancer biology to conservation genetics. Existing model systems, while valuable, lack the direct comparisons between otherwise similar, naturally coexisting sexual and asexual organisms needed to understand sex. The New Zealand snail *Potamopyrgus antipodarum* uniquely allows such comparisons. We present its draft genome assembly, which will enable direct characterization of the causes and consequences of sex. We also discovered a recent whole-genome duplication (WGD). WGDs are significant evolutionary events that are also not well understood. This WGD might both help explain *P. antipodarum's* unique characteristics and offer new avenues for WGD research.

## Introduction

The overwhelming predominance of sexual reproduction in eukaryotes has fascinated biologists for at least 150 years, ever since Charles Darwin (1862) pointed out that any explanation for why so many organisms use sex instead of parthenogenesis was “hidden in darkness”. While we have since learned much about *how* organisms sexually reproduce (reviewed in Ohkura 2015; Bolcun-Filas and Handel 2018), the *why* remains a remarkably persistent evolutionary problem (Zimmer and Emlen 2013; Thomas et al. 2019; Liegeois et al. 2023; Yadav et al. 2023; Miller et al. 2024).

In principle, it should be simple to identify the drivers behind the maintenance of sexual reproduction by comparing factors thought to be related to the advantages of sex (e.g. frequency of intense parasitism, extent of resource limitation) across individuals, populations, or lineages that differ in reproductive mode. In practice, these comparisons are very challenging, in large part because polymorphism for sexual and asexual reproduction in otherwise similar and coexisting individuals is rare in natural populations (Neiman et al. 2018; Miller et al. 2024). The absence of such polymorphism means that the maintenance of sex is not at stake and implies that studies of systems that do not feature conspecific, sympatric, and otherwise similar sexual and asexual individuals are only indirectly applicable to the sex question (Neiman et al. 2018).

### *Potamopyrgus antipodarum* is a Powerful Model for the Study of (A)sex in Nature

Populations of the ancestrally sexual and dioecious Aotearoa New Zealand (hereafter “New Zealand”) freshwater snail *Potamopyrgus antipodarum* are characterized by the coexistence of phenotypically indistinguishable obligate sexuals and obligate asexuals (Lively 1987). Their co-occurrence enables direct comparisons between sexual and asexual individuals and lineages and sexually and asexually propagated genomes. The relative frequency of sexual versus asexual *P. antipodarum* is temporally stable within lakes but varies widely across lakes (Lively and Jokela 2002), enabling the population comparisons required to identify environmental factors contributing to intraspecific maintenance of reproductive mode polymorphism (e.g. Lively 1987; Neiman et al. 2005; Vergara et al. 2014).

Asexual lineages of *P. antipodarum* have been derived on many separate occasions from sexual *P. antipodarum* (Dybdahl and Lively 1995; Paczesniak et al. 2013; McElroy et al. 2021), allowing us to treat each distinct lineage as a replicated test of asexuality. The *P. antipodarum* system is especially unusual in that the origin of asexual lineages does not appear to be associated with hybridization (Phillips and Lambert 1989; Dybdahl and Lively 1995; Neiman et al. 2018). This phenomenon suggests an intrinsic predisposition toward transitions to asexual reproduction (reviewed in Neiman et al. 2014).

*Potamopyrgus antipodarum* is therefore especially well-suited to address major outstanding questions in biology regarding the genetic and genomic factors driving transitions to obligate asexual reproduction, the role of genetic and genomic mechanisms in the maintenance of sexual reproduction, and the genomic consequences of asexual reproduction. *Potamopyrgus antipodarum* is also a textbook example of host–parasite coevolution (Lively 1987; Zimmer and Emlen 2013; Bankers et al. 2017) and is used as a model for ecotoxicology (Geiß et al. 2017; Alonso 2023), mitochondrial–nuclear coevolution (Sharbrough et al. 2017, 2018, 2023; Greimann et al. 2020; Neiman and Sharbrough 2023), invasion biology (Donne et al. 2020, 2022; Geist et al. 2022; Haubrock et al. 2022), and polyploidy (Krois et al. 2013; Neiman et al. 2013; McElroy et al. 2021), indicating that the resources described here will be of wide use.

**Fig. 1.**
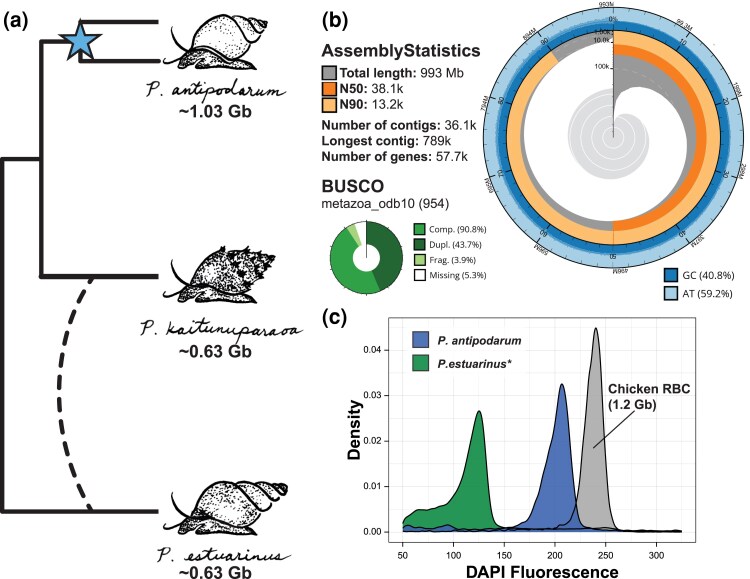
The genome assembly of *P. antipodarum*. a) Cladogram depicting species relationships among *Potamopyrgus* species. Below each species name is the approximate haploid genome size estimated from flow cytometry. The two branches leading to *P. antipodarum* represent the apparent WGD event (blue star) within the species. The dashed line represents putative introgression inferred between *P. kaitunuparaoa* and *P. estuarinus*. See Fields et al. (2024) and Fig. S6 for additional details on this introgression, which is not the main focus of this paper. b) Snail plot describing assembly statistics for the “uncollapsed” *P. antipodarum* contigs. The snail plot in the center is divided into 1,000 size-ordered bins, with each bin representing 0.1% of the assembly. Sequence length distribution is shown in dark gray, with the radius scaled to the longest sequence (shown in red). The N50 sequence length (orange) and N90 sequence length (pale orange) are represented by the arcs in the center of the plot. The cumulative sequence count is represented by the pale gray spiral, with white scale lines showing orders of magnitude. GC and AT percentages are reflected by the dark blue and pale blue areas, respectively, around the outside of the plot. BUSCO marker summary statistics are shown in the bottom left. c) Estimated genome size of *P. antipodarum* (blue/center peak) and *P. estuarinus* (green/left peak) from DAPI-stained nuclei, compared to chicken red blood cells (grey/right peak). Asterisk refers to a similar genome size for *P. kaitunuparaoa*, separately estimated using propidium iodide (data not shown).

Here, we report the first draft genome assembly for *P. antipodarum* (Fig. 1). By comparing this genome to the recently sequenced genomes of fellow congeners *Potamopyrgus kaitunuparaoa* and *Potamopyrgus estuarinus* (Fields et al. 2024), we also uncover unexpected and striking evidence for a recent and still-resolving whole-genome duplication (WGD) event that provides a unique opportunity to study the ongoing process of rediploidization and may help explain the high frequency of transitions to obligate asexual reproduction in this species. The availability of a new high-quality molluscan genome assembly will also be of broad interest and utility to the wider community of molluscan genomics researchers (Davison and Neiman 2021).

## Results

We used four different genomic sequencing technologies and RNA sequencing to produce the first-ever high-quality reference genome assembly and annotation for *P. antipodarum*. This diversity of data was necessary because of the high complexity of the *P. antipodarum* genome, which, as we document below, is largely a consequence of a relatively recent WGD event (Fig. 1a). Accordingly, we varied genome assembly parameters to produce two distinct genomic resources based on these data, both of which will be of wide utility for the *Potamopyrgus* research community: (i) a more biologically realistic but substantially more fragmented set of “uncollapsed” contigs that have not been scaffolded so as to best separate the duplicated regions of the genome, and (ii) a computationally haploidized set of “collapsed” scaffolds that provides large-scale contiguity and simplicity at the expense of structural reality.

### Assembly and Annotation of the *P. antipodarum* Genome

#### Assembly and Gene Annotation

Our uncollapsed *P. antipodarum* assembly had a contig N50 of 38.1 kb and a total length of 993 Mb (Fig. 1b, Fig. S1a). The collapsed version of our *P. antipodarum* assembly had a scaffold N50 of 1.14 Mb and a total length of 543 Mb (Fig. S1b). In total, we annotated 57,703 protein-coding genes in the uncollapsed contigs, 79.0% more genes than the *P. estuarinus* assembly (32,237) and 90.4% more genes than the (relatively incomplete) *P. kaitunuparaoa* assembly (30,310) (Fields et al. 2024). In agreement with the uncollapsed assembly, DAPI-based flow cytometry indicated that *P. antipodarum* (∼2.06 pg) has a substantially larger (1.6 times, or 61%, larger) nuclear genome than *P. estuarinus* (∼1.26 pg) (Fig. 1c, Fig. S2) and *P. kaitunuparaoa* (Fields et al. 2024; Neiman et al. 2025). Additional details about both assemblies and their annotations are provided in the Supplementary Information (Fig. S3), and all materials have been made publicly available (see Data Availability statement).

#### Meiosis Gene Inventory

**Fig. 2.**
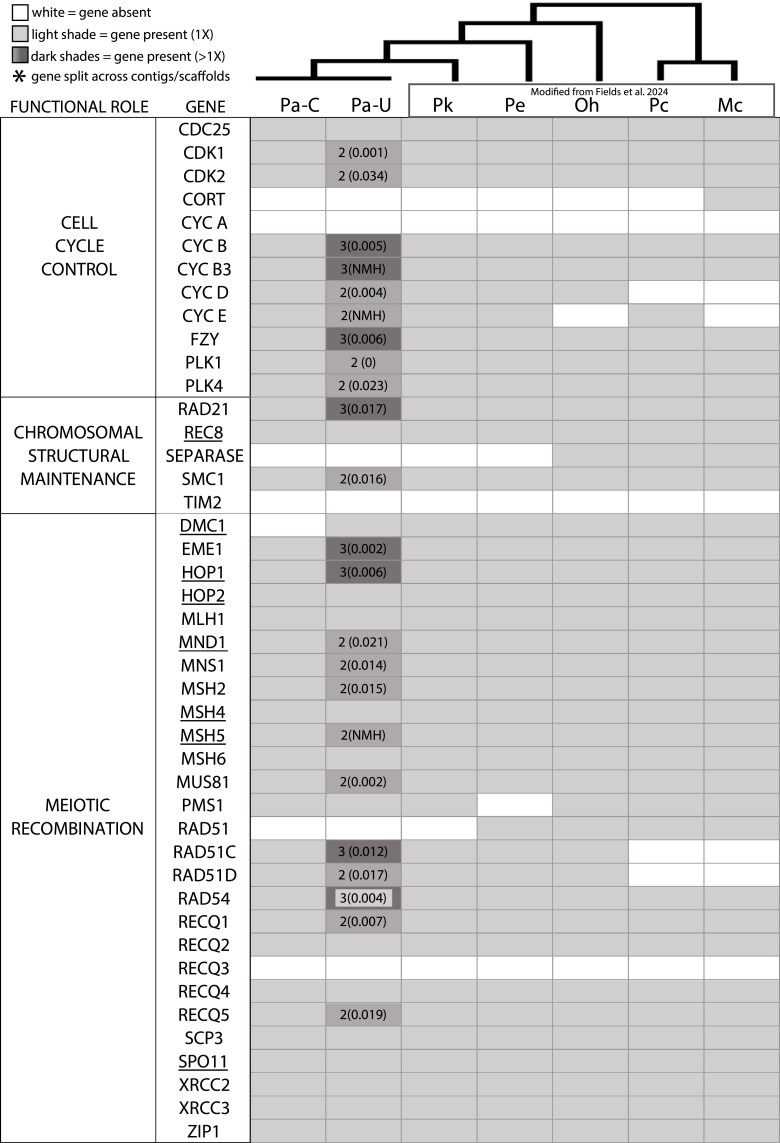
Gene inventory for 44 meiosis genes in 6 gastropod species. Meiosis gene presence and absence, including data from this study and Fields et al. (2024). *Pa* = *P. antipodarum* (the “C” and “U” represent the collapsed and uncollapsed genome assemblies, respectively); *Pk* = *P. kaitunuparaoa*; *Pe* = *P. estuarinus*; *Oh* = *Oncomelania hupensis*; *Pc* = *Pomacea canaliculata*; *Mc* = *Marisa cornuarietis*. For each cell, the number to the left of the parentheses indicates the number of copies present when the gene is at least 80% of the length of the query gene. The number inside the parentheses is the nucleotide p-distance of the mean nucleotide difference between the duplicated copies from the MAKER annotation. NMH = No MAKER hits (these genes were not recovered by the MAKER annotation but were discovered by BLAST and miniprot). A gene is present in each genome assembly if a box is shaded; if the shaded box is empty, the gene is present in a single copy. A gene is absent in each genome assembly if a box is white (unshaded). Bolded and underlined items in the “GENE” column represent genes that are meiosis-specific. The “FUNCTIONAL ROLE” column describes the meiotic function of each gene.

The presence of intact and complete meiosis-specific genes in the genome of an organism is consistent with its ability to engage in meiosis, and thus, sex (Schurko and Logsdon 2008). We compared the meiosis gene repertoire of *P. antipodarum* to those of its congeners, *P. estuarinus* and *P. kaitunuparaoa*, and found that both the collapsed and uncollapsed *P*. *antipodarum* genome assemblies contain the same complement of meiosis genes as the other two species (38 of the 44 queried meiosis genes, Fig. 2). The only exception, *DMC1*, was present in a single copy in the uncollapsed contigs but was absent from the collapsed scaffolds. Every gene absence in the uncollapsed *P*. *antipodarum* assembly was mirrored in *P*. *estuarinus* and *P*. *kaitunuparaoa* (Fields et al. 2024), likely indicating true absence as opposed to an artifact of mis/nonassembly. Three meiosis genes (*RECQ3*, *TIM2*, and *CycA*) were absent from all three species, potentially indicating that these genes are not necessary for meiosis in these caenogastropods. Of the 38 meiosis genes found in the uncollapsed assembly, 23 (60%) were found to exist in multiple copies (single copy = 15/38; double copy = 15/38; triple copy = 8/38). The duplicated genes are nonidentical to one another, with one exception (*PLK1*), and have a nucleotide p-distance distribution ranging from 0 to 0.034 (*x̄* = 0.011; SD = 0.009).

#### Transposable Element Discovery

We annotated TEs in the *P. antipodarum* genome using a variety of approaches (see Supplementary Information for details). In brief, RepeatModeler (using RepeatScout/RECON) found 2,294 repetitive element families, and the LTRPipeline found an additional 253 families, of which 122 were removed in clustering for redundant sequences, for a total of 2,425 repetitive element families. We curated these repetitive element families with the automated pipeline MCHelper, resulting in 957 classified transposable element (TE) families (Table S1, and see Supplementary Information for description of TE classifications).

### Evidence for a Recent WGD and Post-WGD Evolution in *P. antipodarum* Genome

#### Allele Sequencing Depth at Heterozygous Sites

**Fig. 3.**
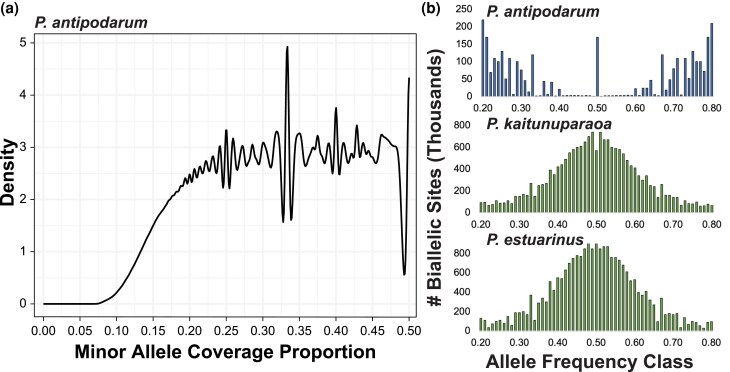
Allelic depth and site-frequency spectra do not meet expectations for diploidy in *P. antipodarum*. a) Density plot summarizing the relative coverage proportion of the minor allele at heterozygous positions, identified in the collapsed assembly. The notable peaks at 0.25 (tetraploidy) and 0.33 (triploidy) indicate that the *P. antipodarum* genome does not fit the typical expectations of diploidy. b) Whole-genome site-frequency spectra for *top*—*P. antipodarum* reads (Alex Yellow) mapped to the uncollapsed *P. antipodarum* assembly, *middle*—*P. estuarinus* reads mapped to *P. estuarinus* genome, and *bottom*—*P. kaitunuparaoa* reads mapped to *P. kaitunuparaoa* genome.

Assuming unbiased sequencing, heterozygous sites are expected to exhibit approximately equal depth of coverage in a diploid (i.e. A:B). By contrast, heterozygous sites in triploids are expected to reflect a 2:1 or 1:2 coverage ratio (i.e. AA:B and A:BB), while tetraploids could, in principle, display the full range of 1:3, 1:1, and 3:1 coverage ratios (i.e. AAA:B; AA:BB; and A:BBB). This expectation allowed a straightforward test of diploidy using the relative depth of coverage for each allele at heterozygous sites, revealing substantial evidence of nonequal coverage across the *P. antipodarum* genome (Fig. 3a). While many sites across the genome fit the null diploid expectation (i.e. 362,125/3,438,187, ∼10.5% of heterozygous sites exhibited minor allele frequencies between 0.45 and 0.5), a large fraction of the genome nevertheless exhibited the 1:2 (2:1) coverage pattern (378,587/3,438,187, ∼11.0% of heterozygous sites exhibited minor allele frequencies between 0.305 and 0.355) or the 1:3 (3:1) coverage pattern (435,622/3,438,187, ∼12.7% of heterozygous sites exhibited minor allele frequencies between 0.225 and 0.275). This distribution of sequencing coverage is not compatible with pure diploidy but instead indicates a more complex genome structure.

#### Site-Frequency Spectrum Analysis

We next estimated genome copy number *in silico* using nQuire (Weiß et al. 2018), leveraging the same data as for the allele depth analysis. The collapsed *P*. *antipodarum* assembly contained 1.59 M high-quality biallelic sites (0.16% of the genome assembly), the *P. estuarinus* assembly contained 11.50 M biallelic sites (2.23% of the assembly), and the *P. kaitunuparaoa* assembly contained 9.32 M biallelic sites (1.56% of the assembly) (Fig. 3b). Alleles at biallelic sites are expected to exhibit a unimodal frequency distribution centered at 0.5 in diploids, while triploids are expected to have peaks at 0.33 and 0.66, and tetraploids are expected to have a trimodal allele frequency distribution with peaks at 0.25, 0.5, and 0.75. We evaluated these distributions using log-likelihoods (1/*Δ*log*L*) for each model comparison, whereby the larger the number, the better the model fits the data. Diploid relatives *P. estuarinus* (1/*Δ*log*L*: diploid = 5.0; triploid = 0.10; tetraploid = 0.01) and *P. kaitunuparaoa* (1/*Δ*log*L*: diploid = 5.0; triploid = 0.14; tetraploid = 0.02) both displayed SFS that best fit a diploid model, whereas *P. antipodarum* (1/*Δ*log*L*: diploid = 0.53; triploid = 0.91; tetraploid = 10.0) had an SFS that best fit a tetraploid model (Fig. 3b). The unexpectedly complex distribution observed in the *P*. *antipodarum* assembly prompted us to estimate the SFSs for each of the 100 scaffolds with the highest number of biallelic sites. Of these, 16 scaffolds best fit a diploid model, two best fit a triploid model, and 81 best fit a tetraploid model (Fig. S4; Table S2). One contig (tig00149948) was an equally good fit for the triploid and tetraploid models (1/*Δ*log*L*: diploid = 0.003; 1/*Δ*log*L*: triploid = 0.01; tetraploid = 0.01). By contrast, all 100 scaffolds with the highest number of biallelic sites in both the *P. estuarinus* and *P. kaitunuparaoa* assemblies best fit a diploid model (Tables S3 and S4).

#### Duplicate Gene Content and Evolutionary History

**Fig. 4.**
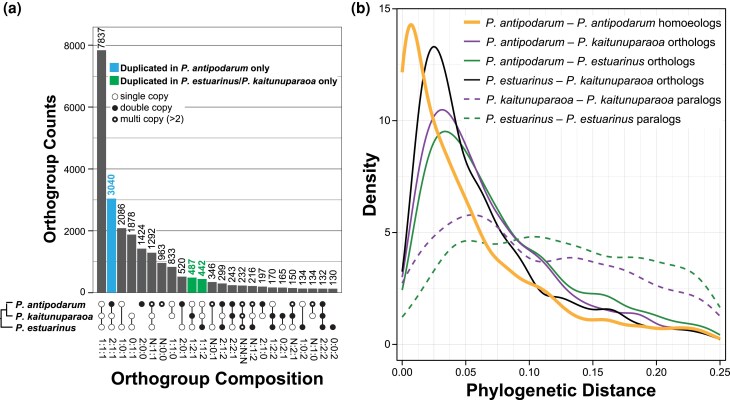
Duplicate gene content and phylogenetic evidence of WGD. a) Upset plot describing orthologous gene group composition in *P. antipodarum*, *P. kaitunuparaoa*, and *P. estuarinus*. *Top—*Histogram of orthogroup number within bins. The blue column represents the 2:1:1 orthogroup bin, for which *P. antipodarum* has two gene copies, while *P. kaitunuparaoa* and *P. estuarinus* have only a single copy. Green columns represent analogous orthogroups in which *P. kaitunuparaoa* and *P. estuarinus* have double-copy genes, but the other species only have a single copy. *Bottom*—Orthogroup bins describing gene content in which white circles represent single-copy genes, black circles represent double-copy genes, and black circles encircling white circles represent multi-copy (i.e. >2) genes. Absence of a circle indicates the absence of a gene from that particular species. The full distribution is available in Fig. S5. b) Density plots of pairwise phylogenetic distance (i.e. patristic distance) from quartet trees (i.e. 2:1:1; 1:2:1; and 1:1:2 orthogroup bins). The solid orange line refers to the phylogenetic distance between gene duplicates specific to *P. antipodarum*. The solid purple line represents the mean phylogenetic distance of both *P. antipodarum* gene copies to *P. kaitunuparaoa.* The solid green line represents the mean phylogenetic distance of both *P. antipodarum* gene copies to *P. estuarinus.* The dashed purple line represents phylogenetic distance between *P. kaitunuparaoa*-specific gene duplicates (1:2:1 orthogroup bin). The dashed green line represents phylogenetic distance between *P. estuarinus*-specific gene duplicates (1:1:2 orthogroup bin).

One of the hallmarks of WGDs (versus, for example, repeat expansion or structural rearrangements) is the instantaneous doubling of gene content across the genome. We first evaluated whether any of the three *Potamopyrgus* species fit this expectation of WGD by using OrthoFinder to infer genome-wide homology among the *P. antipodarum* (uncollapsed), *P. estuarinus*, and *P. kaitunuparaoa* proteomes. The OrthoFinder run produced 25,380 homologous groups containing >1 gene (orthogroups, Fig. S5), 7,837 (30.9%) of which were single-copy in all three species (Fig. 4a). The next most prevalent pattern among orthogroups was for genes that were double copy in *P. antipodarum* but single copy in the other two species (*n* = 3,040, 12.0%). This group was more than six times larger than the number of analogous orthogroups in which two gene copies were represented by *P. kaitunuparaoa* (487 orthogroups) or by *P. estuarinus* (442 orthogroups) but only a single copy from each of the other two species (FET Odds Ratio: 6.96; 95% CI: 6.31 to 7.69; *P* < 0.0001).

We inferred gene trees for both the triplet orthogroups (i.e. 1:1:1) and all possible quartets (i.e. 2:1:1; 1:2:1; and 1:2:2) to estimate phylogenetic distance between gene duplicates. These gene trees (Fig. S6) revealed two patterns: (i) duplicate genes in *P. antipodarum* are much more closely related to each other than either is to *P. kaitunuparaoa* or to *P. estuarinus* (Table 1, Fig. 4b, Table S5), and (ii) all pairs of duplicate genes in *P. antipodarum* appear to have originated around the same time (peak divergence = 0.0070 substitutions/site; Fig. 4b). Neither pattern applies to *P. kaitunuparaoa* or *P. estuarinus* paralogs. This finding indicates that the gene duplicates (hereafter homoeologs) found in the *P. antipodarum* uncollapsed contigs appear to have originated from a WGD, whereas the paralogs present in the other two species reflect the background rate of individual gene duplication. Notably, divergence between *P. antipodarum* and the other *Potamopyrgus species* (peak divergence *P. antipodarum–P. kaitunuparaoa* = 0.032 substitutions/site; peak divergence *P. antipodarum–P. estuarinus* = 0.034 substitutions/site) is substantially greater than the divergence between the *P. antipodarum* homoeologs (Table 1, Fig. 4b). The most clear evidence for this pattern comes from the divergence estimates obtained from the 2:1:1 class of genes (*n* = 3,040): *P. antipodarum* homoeolog divergences for these genes are ∼80% lower than the divergence estimates between *P. antipodarum* and either *P. kaitunuparaoa or P. estuarinus* (0.007 versus 0.033, Fig. 4b). This finding is a clear indicator that the WGD event occurred well after the split between *P. antipodarum* and *P. kaitunuparaoa.*

**Table 1:** Gene tree summary statistics

Orthogroup bin	Median Paralog Divergence (95% CI)^a^	Median *P. antipodarum–P. kaitunuparaoa* Divergence (95% CI)	Median *P. antipodarum–P. estuarinus* Divergence (95% CI)	Median *P. kaitunuparaoa–P. estuarinus* Divergence (95% CI)
*Double-copy genes*				
2:1:1^b^ (*n* = 3,040)	0.038 (0.000 to 0.522)	0.062 (0.007 to 0.688)	0.063 (0.008 to 0.608)	0.065 (0.008 to 0.671)
1:2:1^c^ (*n* = 487)	0.219 (0.000 to 34.58)	0.173 (0.012 to 17.33)	0.073 (0.010 to 1.82)	0.169 (0.016 to 17.33)
1:1:2^d^ (*n* = 442)	0.312 (0.022 to 34.54)	0.067 (0.008 to 1.18)	0.216 (0.027 to 17.28)	0.216 (0.027 to 17.28)
**Concatenated 2:1:1**^e^	**0.054**	**0.072**	**0.079**	**0.063**
*Single-copy genes*				
1:1:1^f^ (*n* = 7,837)	N/A	0.040 (0.004 to 0.688)	0.046 (0.006 to 0.669)	0.040 (0.006 to 0.541)
**Concatenated 1:1:1**^g^	**N/A**	**0.053** (**0.052 to 0.054)**	**0.057** (**0.057 to 0.058)**	**0.052** (**0.052 to 0.053)**

Bold text to distinguish concatenated values.

^a^95% confidence intervals were determined from the value of the 2.5th and 97.5th percentiles of patristic distances from all trees present in the orthogroup bin.

^b^All orthogroups in this bin contain two *P. antipodarum* sequences and one sequence from each of the other two species. Paralog divergence here refers to the total patristic distance from one *P. antipodarum* copy to the other. Divergence from the other species was estimated as the mean between the two branches for each comparison.

^c^All orthogroups in this bin contain two *P. kaitunuparaoa* sequences and one sequence from each of the other two species. Paralog divergence here refers to the total patristic distance from one *P. kaitunuparaoa* copy to the other. Divergence from the other species was estimated as the mean between the two branches for each comparison.

^d^All orthogroups in this bin contain two *P. estuarinus* sequences and one sequence from each of the other two species. Paralog divergence here refers to the total patristic distance from one *P. estuarinus* copy to the other. Divergence from the other species was estimated as the mean between the two branches for each comparison.

^e^All 2:1:1 orthogroups were concatenated in this phylogenetic analysis. Variance estimates are not available from bootstrap replicates produced by RAxML.

^f^All orthogroups in this bin contain a single sequence from each species. Because only a single unrooted tree topology is possible for a tree with three taxa, only branch lengths were estimated using PhyML in this analysis.

^g^All 1:1:1 orthogroups were concatenated for this phylogenetic analysis. 95% CI estimates of patristic distances are from 100 bootstrap replicates in PhyML.

#### Conservation of Synteny of Gene Duplicates

**Fig. 5.**
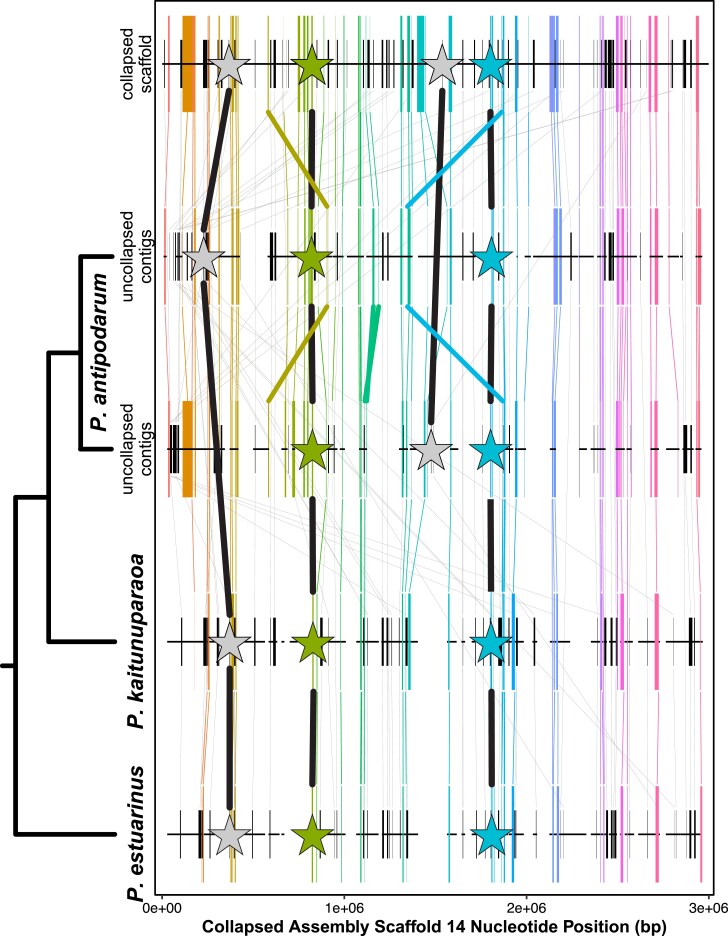
Microsynteny of duplicated and single-copy genes in the *P. antipodarum* genome. Orthologous genes mapped along Scaffold 14 of the collapsed assembly, along with their corresponding positions on the uncollapsed contigs and in the *P. kaitunuparaoa* and *P. estuarinus* assemblies. Genes that were double copy in *P. antipodarum* uncollapsed contigs but single copy in all other assemblies (i.e. 2:1:1 orthogroup bins, *n* = 57) are represented by tall colored boxes, with the width reflecting the length of the gene. Genes that are single copy in all species (i.e. 1:1:1 orthogroup bins, *n* = 62) are represented by short black boxes, with the width of the box representing gene length. Lines connecting orthologs are colored for 2:1:1 orthogroups and grey for 1:1:1 orthogroups. Large stars represent BUSCO genes (connected by black lines), with colored stars being 2:1:1 genes and grey stars being 1:1:1 genes. Thick colored lines reflect rearrangements found in 2:1:1 genes.

Another hallmark of WGD, and a useful tool for determining when these genome duplication events occurred, is the extent of conserved gene collinearity (i.e. synteny) across groups of duplicated genes arrayed along a chromosome (Dehal and Boore 2005). The potential for extensive recombination-derived structural rearrangements in the wake of WGD (Bohutínská et al. 2021; Hämälä et al. 2024) can make this pattern challenging to identify, but even genome-wide blocks of microsynteny (i.e. 10 to 50 genes) can provide powerful evidence of homoeology (Conover et al. 2021). Here, we leveraged the collapsed *P. antipodarum* scaffolds to serve as anchor points with which to compare synteny in the uncollapsed contigs, with *P. kaitunuparaoa* and *P. estuarinus* as outgroup references. We found evidence of global microsynteny across *P. antipodarum* homoeologs, interrupted by complex structural rearrangements (Fig. S7). As an exemplar, we performed an in-depth characterization of microsynteny on Scaffold 14 from the collapsed assembly (Fig. 5), as it featured the longest block of 2:1:1 orthogroups along a single scaffold (*n* = 57). In this 3-Mbp genomic region, we can simultaneously observe the maintenance of synteny among homoeologous gene pairs, individual gene loss, and even structural variation across subgenomes. There was perfect microsynteny across the genus for 53/57 (93%) of 2:1:1 orthogroups, whereas only 45/62 (73%) of single-copy orthogroups exhibited conserved microsynteny with the other *Potamopyrgus* species. The greater degree of structural rearrangement among single-copy genes potentially reflects the mechanism of gene loss (i.e. via ectopic recombination). Overall, these synteny data provide clear evidence that a WGD event followed by complex and extensive post-WGD chromosomal rearrangements underpins genome evolution in *P. antipodarum*.

#### TE Abundance Does Not Explain the Increase in *P. antipodarum* Genome Size

**Fig. 6.**
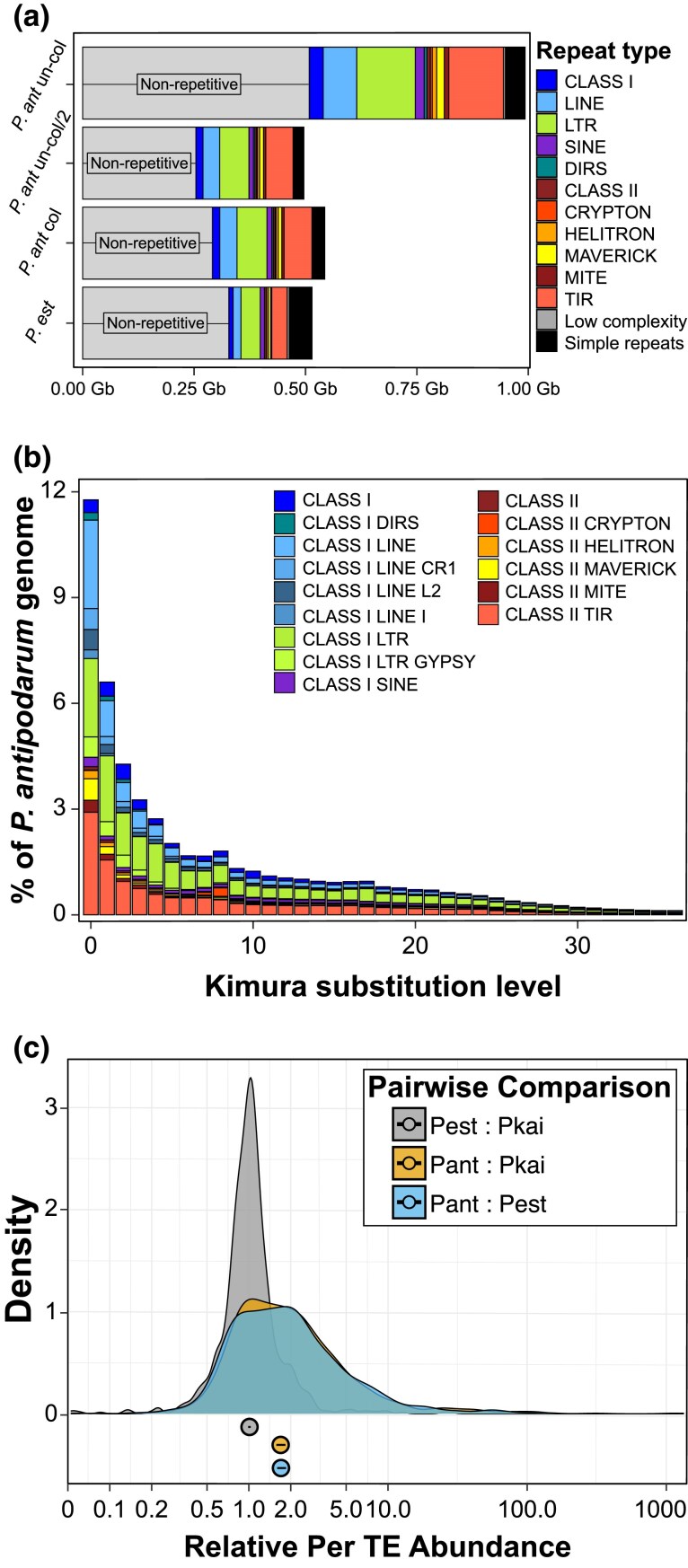
TE expansion in the *P. antipodarum* genome. a) Comparison of repetitive sequence content across *P. antipodarum* genome assembly versions and *P. estuarinus* based on RepeatMasker results using the curated *P. antipodarum* TE library. Genome size for each assembly is reflected in the *X* axis. Repeat types (classes and major divisions of TE types) are color-coded, along with “Non-repetitive” genomic content (i.e. not masked by any repeat category). Genome assemblies are as such, “*P. ant* un-col”: uncollapsed (contigs) genome assembly for *P. antipodarum*; “*P. ant* un-col/2”: results for the uncollapsed genome assembly for *P. antipodarum* divided by 2; “*P. ant* col”: collapsed (scaffold) assembly for *P. antipodarum*; and “*P. est*”: *P. estuarinus* genome assembly. b) Repeat landscape for *P. antipodarum*, depicting past TE activity in the genome. *X* axis: Kimura substitution level relative to the family consensus sequence, reflective of TE family age. Younger TEs (i.e. more recently inserted into the genome) have lower levels of pairwise divergence compared to older TEs. *Y* axis: percent of the genome made up by each TE group. c) Relative abundance of TEs in pairwise species comparisons. Copy numbers estimated from read mapping depth via DeviaTE for 942 TE families, shown here as ratios of copy numbers between species, e.g. for a “relative copy number” of 1.0, the two species in comparison have identical copy numbers. Three pairwise comparisons, e.g. *P. antipodarum*: *P. estuarinus* (Pant: Pest) and *P. antipodarum: P. kaitunuparaoa* (Pant: Pkai), are shown as density plots of 942 relative values, with median and 95% CI shown below. Example highlighted in text, DNA transposon rnd-5_family-12008, copy numbers: Pkai = 4.306, Pest = 6.943, Pant = 197.091; relative values: Pest:Pkai = 1.6, Pant:Pkai = 45.8, Pant:Pest = 28.4.

To rule out the possibility that TE expansion is responsible for the larger genome size of *P. antipodarum*, we compared the TE proportion and total genomic TE content across three *Potamopyrgus* species. DNApipeTE estimates of genomic TE content indicated that *P. antipodarum* harbors more TEs than its congeners, with 42% of genomic reads mapping back to putative TEs versus ∼25% for *P. estuarinus* and *P. kaitunuparaoa* (Table S6). The RepeatMasker results are overtly similar, with TEs representing 45.6% of the *P. antipodarum* genome and only 26.1% of the *P. estuarinus* genome (Table 2). The outcome of the analysis of the masked and collapsed version of the *P. antipodarum* genome was virtually identical, with 42.8% TEs (Fig. 6a). The congruencies between our assembly-based and read-based measures of TE abundance, including the two levels of assembly for the *P. antipodarum* genome assembly, indicate that we accurately captured the proportional differences in TE load across *Potamopyrgus* species.

**Table 2:** Transposable element content in *P. antipodarum* compared to other *Potamopyrgus* species

Repeat classification		DNApipeTE			RepeatMasker	
		*P. antipodarum*	*P. estuarinus*	*P. kaitunuparaoa*	*P. antipodarum*	*P. estuarinus*
Class I						
	LTR	2.54%	0.01%	1.68%	13.23%	8.43%
	LINE	4.90%	2.28%	1.91%	7.58%	3.42%
	SINE	0.40%	0.77%	0.37%	2.02%	1.85%
	DIRS	n/a	n/a	n/a	0.64%	0.11%
	other	n/a	n/a	n/a	2.52%	1.71%
Class II						
	TIR	3.66%	3.19%	3.02%	13.25%	7.12%
	Maverick	n/a	n/a	n/a	1.72%	1.04%
	Helitron	0.36%	0.21%	0.33%	1.01%	0.73%
	Crypton	n/a	n/a	n/a	0.43%	0.08%
	other	n/a	n/a	n/a	0.75%	0.77%
Other repeats						
	Low complexity	0.20%	0.48%	0.56%	0.42%	0.94%
	Simple	2.26%	3.89%	4.22%	4.45%	9.86%
	other	0.34%	0.95%	1.04%	n/a	n/a
Unclassified		30.16%	17.82%	17.24%	n/a	n/a
**Total TE**		**42.02%**	**25**.**75%**	**24**.**56%**	**43**.**15%**	**25**.**26%**
**Total repeat**		**44.83%**	**31**.**06%**	**30**.**37%**	**48**.**02%**	**36**.**06%**

Bold text for emphasis on total values.

Although our results revealed a considerably larger fraction of TEs in *P. antipodarum* relative to two congeneric species, the genome size disparities between *P. antipodarum* and *P. estuarinus* cannot be explained by TEs alone. Notably, the total bp of TEs in the *P. antipodarum* genome assembly was about 3.3× higher than in the *P. estuarinus* assembly, while the nonrepetitive portion of the *P. antipodarum* genome was still about 1.5× greater than that of *P. estuarinus*, closely matching the 1.8× more predicted genes.

#### Recent “Bursts” of TE Activity in *P. antipodarum* Genome

Over 20% of the *P. antipodarum* genome is made up of TEs with within-family divergences of 2% or less (Fig. 6b). The proportion of TE sequence belonging to the 0% to 2% divergence bins was more than double the proportion of TE sequence in the 3% to 5% divergence bins, demonstrating a global increase in TEs across all classes. Read-mapping approaches also revealed higher abundances of TEs (on a per-element basis) in *P. antipodarum* relative to *P. estuarinus* and *P. kaitunuparaoa* (Fig. 6c). While some elements demonstrated no notable difference in copy number across species (see example in Supplementary Information), others exhibited substantial increases in copy number specific to *P. antipodarum* (Fig. S8). Overall, these comparative results demonstrate a systematic increase in TE abundance in *P. antipodarum* relative to its close relatives, and in one case, a likely active DIRS retrotransposon exclusively found in *P. antipodarum* (Fig. S9, and see supplementary text for details).

We next asked whether there existed dramatic differences in copy number and relative abundance of particular TE families across species as an indicator of “bursts” of TE activity. For this analysis, we applied an arbitrary threshold of differences in copy number of at least 100 and at least 5× difference in relative abundance. For *P. antipodarum* versus *P. estuarinus*, 24 TEs met these criteria, while 27 TEs met the criteria for *P. antipodarum* versus *P. kaitunuparaoa* (with 20 in common across these 2 pairs). There were no other comparisons that reached these thresholds across any of the other pairwise species combinations, with the exception of one TE family in *P. estuarinus* compared to *P. kaitunuparaoa* (110.4 copies in *P. antipodarum*, 284.2 copies in *P. estuarinus*, and 28.7 copies in *P. kaitunuparaoa*). This result demonstrates that the recent accumulation of TEs is characteristic only of *P. antipodarum*.

We also examined the RepeatMasker results to identify elements with likely ongoing activity to ask whether these elements are more abundant in *P. antipodarum*. The criteria we considered were the sequence divergence between copies of TEs of the same family and “completeness” of copies in the *P. antipodarum* genome assembly (i.e. how long is the average copy length compared to the reference length of the TE?). The element with the lowest Kimura substitution level (0.3%) was a *Mariner* DNA transposon that also exhibited evidence of a burst of activity in *P. antipodarum* compared to its congeners, with nearly 200 more copies (Fig. S10, and see Supplementary Information for additional details). The TE with the longest average genomic insertion (8,024 bp versus 13,140 bp reference length) was classified as a *Maverick* DNA transposon, which also fit our criteria for burst. This *Maverick* element accounts for about 3.5 Mb of genomic sequence in *P. antipodarum* versus about 0.05 Mb in *P. estuarinus* and 0 bp in *P. kaitunuparaoa* (see Supplementary Information for additional details).

#### Gene Family Increases in *P. antipodarum*

We identified 1,453 orthogroups that met our “expanded” criteria, meaning orthogroups in which *P. antipodarum* had more gene copies than would be expected of WGD alone, and found 26 GO terms significantly enriched (*adjusted P* < 0.05) in the “expanding” gene set (Fig. S11 and see Table S7 for lists of genes in enriched groups). Among the enriched GO categories were several related to cell cycle regulation and DNA repair, including “mitotic chromosome condensation,” “ATP-dependent DNA damage sensor activity,” “double-strand break repair,” “ATP-dependent chromatin remodeler activity,” and “interstrand cross-link repair.” The non-SMC subunits for condensin complexes (chromosome-associated proteins, or CAPs) were among the genes in these orthogroups related to chromosome condensation. We found that each of the three subunits for condensin I (CAP-D2, CAP-G, and CAP-H) and condensin II (CAP-D3, CAP-H2, and CAP-G2) are present at 3 or 4 copies in our genome-wide annotation of the uncollapsed assembly, with the condensin II complex subunits CAP-D3 and CAP-H2 each having four copies.

## Discussion

We produced the first high-quality reference genome assembly for *P. antipodarum*, a New Zealand freshwater snail that has risen to prominence as a model system for the study of sexual reproduction, host-parasite coevolution, genome size variation, and invasion biology. Analysis of this genome assembly also revealed multiple strong lines of evidence for an unexpected and relatively recent WGD unique to *P. antipodarum* and rampant post-WGD activation of TEs.

Together, our analyses point to a scenario in which the differential retention and loss of extra chromosome copies post-WGD has resulted in a mostly tetraploid mosaic genome with a substantial minority of triploid and diploid genomic regions. This complexity is consistent with expectations for rediploidization following a WGD (Bohutínská et al. 2021; Hämälä et al. 2024). The extent to which the redundant genome copies are retained or expunged via random genetic drift or natural selection is an exciting open question. Both selection (Wang et al. 2021) and genetic drift (Johri et al. 2022) have been implicated in the (non)random genomic winnowing that generally occurs after WGD as rediploidization progresses. The variable nuclear genome size, even with respect to diploid sexual *P. antipodarum* (Neiman et al. 2011, 2025), means that these questions can be addressed with comparisons between *P. antipodarum* individuals that differ in nuclear DNA content. Such analyses will illuminate how selection and genetic drift operate under conditions in which excess genomic material has been gained (McGrath et al. 2014; Mandáková and Lysak 2018).

### General Implications of WGD

We unexpectedly discovered multiple strong lines of evidence for a very recent WGD in the ancestor of extant *P. antipodarum*. WGD events are among the most profound mutational changes observed in nature (reviewed in Anatskaya and Vinogradov 2022), with effects ranging from the genome (e.g. Bohutínská et al. 2021; Gonzalo et al. 2023; Mihajlovic et al. 2025) and cell (e.g. Losick and Duhaime 2021; Cadart et al. 2023; Zhang et al. 2023) to the organism (e.g. Pacey et al. 2022; Li et al. 2024; Westermann et al. 2024) and population (e.g. Leitch and Leitch 2008; Landis et al. 2018; Meudt et al. 2021 ; Edgeloe et al. 2022; Parshuram et al. 2023). The genome-wide redundancy that results from WGDs has long been thought to provide the fuel for evolutionary innovation (Ohno 1970; Mihajlovic et al. 2025), with important adaptations, such as seeds (Jiao et al. 2011), flowers (Amborella Genome Project 2013), improvements in visual systems (Parey et al. 2020), and many other morphological and developmental innovations (Soltis and Soltis 2016; Peskin et al. 2020; Zhang et al. 2021; Farhat et al. 2023), owing their origins to WGDs.

Among the most perplexing consequences of WGDs is the seemingly inevitable return to diploidy following genome duplication (Wendel 2015). Indeed, although the vast majority of extant eukaryotes are diploid, almost all have experienced WGD events in their evolutionary past (Wendel 2000; Sémon and Wolfe 2007), including humans (Dehal and Boore 2005). The rate and pattern of rediploidization is therefore of great interest to evolutionary biologists, and much has been learned over recent years, especially with the explosion of plant genomic resources (One Thousand Plant Transcriptomes Initiative 2019; Li et al. 2021; Meudt et al. 2021). Whether these lessons extend to animals is unclear, in large part because only two animal systems (i.e. *Xenopus* [Session et al. 2016] and salmonids [Berthelot et al. 2014; Dimos and Phelps 2023]) have the necessary genomic resources. Comparing a diverse set of independent animal WGDs is therefore critical to identifying the “rules” (if any exist) of postpolyploid genome evolution. Our *P. antipodarum* genome assembly provides a valuable advance in this respect (Davison and Neiman 2021; Liu et al. 2021).

The WGD inferred here appears to have occurred considerably more recently than the split between *P. antipodarum* and the two other *Potamopyrgus* species. Further, while the lack of mutation rate estimates for *Potamopyrgus* and the absence of fossils with which to calibrate phylogenies prevent absolute dating of the WGD event, our relative estimates of the timing of WGD suggest that the *P. antipodarum* genome exhibits patterns consistent with “mesopolyploidy,” a time during which a duplicated genome undergoes re-diploidization. Mesopolyploidy in *P. antipodarum* is substantiated by two central observations. First, the distinct prevalence of single-copy genes (31%) in the genome over double-copy genes (12%) indicates that many, but not all, genes have already returned to single-copy status. Second, a diverse and large survey of *P. antipodarum* and *P. estuarinus* recently confirmed that this WGD does indeed seem to be representative of the former, including the smaller-scale genome-size variation expected as a function of variable rediploidization processes across the species (Neiman et al. 2025). Accordingly, *P. antipodarum* promises to be an exciting setting in which to investigate the tempo and patterns of pseudogenization and recombinative deletion, and their relative contributions to genome downsizing (Crombez et al. 2025).

### Meiosis Genes in the Context of WGD

In the *P*. *antipodarum* genome assembly, 38/44 (86%) of the meiosis genes queried are present in at least one copy, and at least one of those copies is intact. The presence and likely intact function of most of the genes needed to maintain meiosis (Schurko and Logsdon 2008) in the inbred sexual *P. antipodarum* line that we sequenced here, as well as the nearly identical meiosis gene complement in closely related obligately sexual species, are as expected if purifying selection is maintaining gene function required for obligate sex. Some of these duplicated genes have disrupted reading frames or are missing portions of exons, suggestive of rediploidization following the WGD (Dehal and Boore 2005; Sémon and Wolfe 2007; Qiao et al. 2019; Parey et al. 2022). Nevertheless, the fact that all genes present in *P*. *antipodarum* are also present in closely related congeners as well as in more distantly related taxa provides solid evidence that these genes are indeed maintained in *P. antipodarum*.

In contrast to the duplicated pattern seen for other meiosis genes, the meiosis-specific gene *DMC1* is present as a single copy in the uncollapsed *P. antipodarum* genome. This finding suggests that selection might have favored the loss of an extra copy of *DMC1* following WGD. The negative effects imposed by additional *DMC1* copies could be a consequence of nonmutually exclusive mechanisms like dosage sensitivity, increased risk of ectopic recombination, or disruption of interactions with other proteins of the same recombination gene complex (e.g. *DMC1* and *RAD51*). *DMC1* is highly conserved as a single-copy gene across eukaryotes, including closely related gastropod taxa (Fields et al. 2024), indicating strong evolutionary constraint. We cannot completely exclude the hypothesis that the apparent absence of a second copy of *DMC1* in the *P. antipodarum* assembly may actually reflect technical limitations; for example, sequence complexity (e.g. repetitive motifs) could result in the misassembly of regions containing additional *DMC1* copies.

Conspicuous meiosis gene absences included *RECQ3*, *TIM2*, and *CYCA*. The absence of these three genes in the diverse set of Caenogastropod taxa that we surveyed could indicate that their absence is characteristic of this gastropod subclass (Schurko and Logsdon 2008; Schurko et al. 2009). Indeed, these “incomplete” meiotic toolkits seem to be the rule rather than the exception, found in taxa from diatoms (Patil et al. 2015) and choanoflagellates (Carr et al. 2010) to *Trichomonas* (Malik et al. 2007) and many more (Hofstatter et al. 2018; Wilding et al. 2020; Tekle et al. 2022; Catta-Preta et al. 2023).

### TE Proliferation Following WGD

Among the expected consequences of WGD are altered regulation and evolution of TEs (Vicient and Casacuberta 2017; Wendel et al. 2018). In particular, polyploidization has long been thought of as a “genomic shock” that enables bursts of TE activity (McClintock 1984). While polyploids tend to have more TEs than diploid relatives, the mechanisms underlying this phenomenon remain an open question (Parisod et al. 2010; Vicient and Casacuberta 2017), particularly concerning the relative contributions of TE activity and efficacy of natural selection in removing TE insertions in polyploids (Ågren et al. 2016; Baduel et al. 2019; Scarlett et al. 2023). Our analysis of the *P. antipodarum* genome revealed a global increase in TEs relative to its close relatives and evidence of recent bursts of multiple specific TE families.

TEs are important contributors to genome size variation (Kidwell 2002) and may account for dramatic differences in genome size between even closely related species (e.g. Talla et al. 2017; Blommaert et al. 2019; Naville et al. 2019), with expansion of a single TE family capable of rapidly increasing genome size (Wong et al. 2019). Although our results revealed a considerably higher burden of TEs in *P. antipodarum* relative to congeners (e.g. 3.3× more total DNA in genome assemblies), the higher amount (1.5 to 1.8×) of DNA in genes and nonrepetitive genomic regions means that nuclear genome size disparities between *P. antipodarum* and *P. estuarinus* cannot be explained by TEs alone. It is possible that the genic region of the *P. antipodarum* genome is rediploidizing at a higher rate than repetitive regions, which may account for the asymmetry in expanded DNA regions relative to *P. estuarinus*. Post-WGD TE proliferation may also rapidly add substantial quantities of DNA to the genome, driving up the proportional differences in TE abundance. As an example from *P. antipodarum*, we identified a 13 kb *Maverick* DNA transposon with hundreds of more copies in the *P. antipodarum* genome compared to its congeners that alone account for a 3.5 Mb difference in genome assembly length.

Along with this *Maverick* transposon, we identified several other likely active elements representing different TE classes that exhibit signs of activity bursts in *P. antipodarum*, e.g. low sequence divergence across—mostly—complete copies and dramatically greater abundance than in other *Potamopyrgus* species. Population-level and polymorphism data from multiple species of *Potamopyrgus* will allow for further tests of the influence of WGD on TE evolution and for evaluation of whether the WGD might have increased TE activity in *P. antipodarum*. Finally, the apparently recent invasion of some TEs raises important questions about genome stability and susceptibility to TE colonization and mobilization and will allow us to use *P. antipodarum* to explore the influence of asexuality on TE evolution, including direct evaluation of the conflicting expectations regarding how the absence of sex will affect TE proliferation (Hickey 1982; Zeyl et al. 1994; Zeyl and Bell 1996; Arkhipova and Meselson 2000 , 2005; Wright and Finnegan 2001; Nuzhdin and Petrov 2003; Dolgin and Charlesworth 2006; Schurko et al. 2009; Jaron et al. 2021).

### Could the WGD Underpin the High Levels of Variation and the Evolution of Asexual Reproduction Observed in *P. antipodarum?*

*Potamopyrgus antipodarum* is notable as one of the few natural systems with coexisting and competing obligately sexual and asexual forms (Neiman et al. 2018). While some other animal taxa do experience occasional transitions to asexual reproduction (Otto and Whitton 2000; Lundmark and Saura 2006), most of these cases are tied to hybridization and are observed only rarely (Neiman and Schwander 2011), versus the evidence for many and frequent such transitions in *P. antipodarum* (Fox et al. 1996; Jokela et al. 2003; Paczesniak et al. 2013). *Potamopyrgus antipodarum* is the only species in the large Tateidae family known to have evolved asexual reproduction (Ponder et al. 2019), and there are only two other gastropod taxa for which parthenogenesis has been documented (reviewed in Auld and Jarne 2016). *Potamopyrgus antipodarum* harbors high phenotypic diversity relative to other New Zealand congeners (Winterbourn 1970; Ponder 1988; Haase 2003, 2008), and asexual assemblages of *P. antipodarum* are rich in genetic diversity relative to other naturally occurring asexual populations (Fox et al. 1996; Jokela et al. 2003). This snail also inhabits a remarkably wide range of habitats (Winterbourn 1970; Haase 2008) and has established invasive populations across the world (Geist et al. 2022).

Could a recent WGD help to explain why *P. antipodarum* is so unique? There is a growing body of evidence that WGD can catalyze the evolution of novel phenotypes (recently reviewed in Anatskaya and Vinogradov 2022). We are unaware of studies demonstrating a causal link between WGD and frequent transitions to parthenogenesis, but there are several cases of strong links between WGD and other innovations in key features of reproductive biology (e.g. Soltis and Soltis 2016; Charron et al. 2019). The extra copies of chromosomes and genes that characterize WGD can also provide a means of compensating for some of the hypothesized negative consequences of asexual reproduction (e.g. mutation accumulation, lack of genetic variability) via mutational masking (Otto and Whitton 2000; Conover and Wendel 2022) and as a new source of genetic variation (e.g. Van de Peer et al. 2017; Ren et al. 2018). Moreover, the rediploidization that follows WGD could lead to broad-scale genomic incompatibilities resulting from lineage-specific genome fractionation, from which the only evolutionary escape is asexuality. Thus, WGD may provide a scenario in which asexuality is able to gain a foothold in natural populations. The resources we present here provide a powerful means forward to begin targeted evaluation of whether and how WGD might enable the repeated separate transitions to obligate asexuality that make *P. antipodarum* so remarkable.

### Gene-Family Evolution Associated with Challenges of Polyploidy?

As part of our analysis of the WGD event in *P. antipodarum*, we also tested for functional enrichments in both expanding and contracting gene families to help describe the effects of WGD on the rest of the genome. We found that orthogroups with *P. antipodarum*-specific expansions (i.e. cases in which *P. antipodarum* had relative gene counts of at least 3:1:1 compared to *P. estuarinus* and *P. kaitunuparaoa*) were enriched for GO terms involving DNA maintenance, organization, and segregation, including double-strand break repair, mitotic chromosome condensation, and numerous microtubule-related categories. We also found that, based on our genome-wide annotation of the uncollapsed assembly, *P. antipodarum* possesses 3 to 4 copies of the condensin subunits. As their name implies, condensins are protein complexes (condensin I and condensin II) involved in organizing and segregating chromosomes during cell division (Hirano 2016). This observation is congruent with outcomes from our careful annotation of meiosis-related genes, including three copies each of multiple *CYC* genes and *FZY*, all genes known to be involved in cell-cycle regulation (Fig. 2 and see Swan and Schüpbach 2007; Wolgemuth and Roberts 2010; Bouftas and Wassmann 2019).

These results raise the possibility that *P. antipodarum* evolved compensatory mechanisms to handle expanded DNA content (e.g. during meiosis; Gonzalo et al. 2023) via retention of duplicated genes and/or further gene duplication, though a close examination of the sequences and expression patterns of the genes involved in mitosis and meiosis is a necessary next step to explore this hypothesis. Population-genomic analyses would also permit a more direct evaluation of the possibility that *P. antipodarum* is adapting to a sudden increase in DNA content through selection on cell-division genes. This scenario could be analogous to autopolyploid *Cochlearia officinalis*, whereby such analyses revealed evidence of genomic adaptation to key stressors of doubling DNA content: organization of DNA and maintaining ion homeostasis, with evidence for positive selection on kinetochore proteins and sodium and potassium transporters (Bray et al. 2024). Our GO-enrichment results also indicate that ion transporter gene families, including those related to potassium, have increased their representation in *P. antipodarum*. As ion homeostasis perturbation is a consequence of genome doubling (Chao et al. 2013 ), these genes will also make for interesting follow-up investigations.

From the CAFE results, we identified 287 orthogroups with expansions in *P. antipodarum*, which were enriched for 51 GO-terms (Fig. S11 and Table S7). These results similarly included terms related to DNA maintenance and cell cycle regulation, such as “DNA recombination,” “mitotic cell cycle checkpoint signaling,” “RZZ complex,” “mismatch repair complex,” “chromatin organization,” and “chromosome centromeric region,” as well as eight microtubule-related GO terms.

### Summary and Conclusions

The *P. antipodarum* genome assembly that we have produced represents a unique resource that will be of use to the many scientists interested in a host of important open questions in biology, from the maintenance of sex and the evolution of biological invasions to the consequences of WGD and the genomic drivers of host-parasite coevolution. Our genome assembly will enable downstream work targeting the genomic or genetic regions involved in the frequent transitions to asexual reproduction in *P. antipodarum*. We have also brought together multiple lines of evidence, including some new ways to evaluate genomes for the presence of WGD, to demonstrate a recent WGD in *P. antipodarum* that appears to be “caught in the act” of the process of rediploidization. This discovery opens the door to using different *P. antipodarum* lineages to evaluate the extent to which genomic incompatibilities produced by rediploidization proceeding independently in allopatric lineages could contribute to frequent transitions to asexuality in this system.

## Materials and Methods

To assemble a representative genome sequence of *P. antipodarum*, we generated genomic data from an inbred sexual diploid lineage (“Alex Yellow”) founded by a sexual female collected from Lake Alexandrina in the 1990s. We selected this lineage for the reference genome because it had been inbred for ∼20 years (∼20 to 30 generations; Larkin et al. 2016) and was thus likely to have relatively high homozygosity, facilitating the genome assembly process. This lineage has also been used in other sequencing projects (Neiman et al. 2010; Sharbrough et al. 2018, 2023). We used three additional Alex Yellow individuals for the generation of transcriptomic data: one brooding (reproductive) adult female, one nonbrooding adult female, and one adult male. Detailed methods for assembly and annotation with MAKER and BUSCO (Manni et al. 2021) can be found in the Supplementary Information.

### Evaluating Evidence for a Recent WGD in *P. antipodarum*

#### Genome Size Estimation with Flow Cytometry

We determined the approximate total nuclear genome size using flow cytometry, as described by Neiman et al. (2011). Briefly, flash-frozen *P. antipodarum* and *P. estuarinus* head tissues were ground in a solution containing 0.2 M Tris–HCl (pH 7.5), 4 mM MgCl_2_, 1% Triton X-100, and 4 μg/mL DAPI. This solution was filtered through a 70-micron nylon sheet and assayed on a Beckman-Coulter Quanta SC MPL flow cytometer (Beckman Coulter, Brea, California, United States). Four *P. estuarinus* samples were assayed on 2015 January 27, and 40 *P. antipodarum* Alex Yellow samples were assayed on 2015 February 13. We used the FL1 channel to assess DAPI fluorescence (and thus the total DNA content) of cell nuclei under a UV lamp. At the beginning and end of each flow cytometry run, we calibrated the machine using 20 μL of chicken red blood cells (Lampire Biological Labs, Pipersville, Pennsylvania, United States) treated and filtered in the same manner as the *Potamopyrgus* samples. Each sample was run until a count of at least 10,000 events was achieved.

#### Allele Sequencing Depth at Heterozygous Sites

As a preliminary test of WGD, we designed a mapping experiment to compare relative sequencing coverage of alleles at heterozygous sites. To do this, we mapped Illumina HiSeq reads collected from a single individual female snail to the collapsed assembly (see Supplementary Information) using bwa v0.7.17-r1188 (Li and Durbin 2010) with default parameters. We called variants using GATK v4-4.6.1.0-0. As part of this procedure, we first removed PCR and optical duplicates using the MarkDuplicates function, then called variants, assuming diploidy, using the HaplotypeCaller function. We extracted allele sequencing depth from the DP4 field of the resulting VCF file for every heterozygous site called by GATK4, calculated the proportion of reads covering the minor allele as a fraction of the total depth over the site, and visualized the distribution of these data using the ggplot2 subpackage (Wickham 2011) in R v4.4.2 (R Core Team 2025).

#### Site-Frequency Spectrum Analysis

The distribution of allele frequencies (i.e. site-frequency spectrum; SFS) at biallelic sites can be used to infer genomic copy number and, thus, estimate ploidy *in silico* (Yoshida et al. 2013; Margarido and Heckerman 2015; Zhu et al. 2016; Li et al. 2017; Ranallo-Benavidez et al. 2020; Becher et al. 2022; Gaynor et al. 2024). Here, we used nQuire (Weiß et al. 2018), a bioinformatic tool that estimates intragenomic SFSs, to test for the possibility of a WGD event specific to the *P. antipodarum* lineage. As part of this analysis, we compared the nQuire output from *P. antipodarum* with that obtained from the assemblies of the diploid congeners *P. estuarinus* and *P. kaitunuparaoa* (Fields et al. 2024). Additional details about this analysis are available in the Supplementary Information.

#### Gene-Based Tests for WGD

The outcomes of the analyses described above, coupled with the challenges we experienced during de novo assembly of the *P. antipodarum* genome, led us to hypothesize that *P. antipodarum* had experienced a recent WGD. We evaluated this hypothesis through a series of gene-based analyses: (i) comparative sequence-based intragenomic homology (i.e. the global distribution of paralogs in *P. antipodarum* versus *P. kaitunuparaoa*, and *P. estuarinus*), (ii) phylogeny-based inference of paralog divergence and tree topology, and (iii) synteny-based homology (i.e. collinearity of duplicated genes in *P. antipodarum*). Details of these analyses are provided in the Supplementary Information.

#### Genome-Wide Scan of Changes in Gene Family Size

To investigate broad patterns of gene family expansions between *P. antipodarum* and its congeners, we tested for GO-term enrichment among orthogroups for which *P. antipodarum* had more (“expanded”) genes relative to *P. estuarinus* and *P. kaitunuparaoa*. The expanded orthogroups were defined as those in which *P. antipodarum* had more than double the number of genes than either *P. estuarinus* or *P. kaitunuparaoa* (i.e. ratios of gene counts greater than 2:1:1 in *P. antipodarum*: *P. estuarinus*: *P. kaitunuparaoa*). We used the enricher function in clusterProfiler v4.8.3 (Wu et al. 2021) to test for GO term enrichment, applying a Benjamini–Hochberg false discovery rate for an adjusted *P*-value of 0.05.

We also used CAFE5 v1.1 (Hahn et al. 2005; De Bie et al. 2006; Mendes et al. 2021) to identify significant changes in orthogroup size and cases of gene number increases in *P. antipodarum*. Here, we ran OrthoFinder with genes from the three *Potamopyrgus* species, along with two outgroup gastropods: *Pomacea canaliculata* (proteins from GCF_003073045.1) and *Littorina saxatilis* (proteins from GCA_037325665.1). We then selected orthogroups that fit the following three criteria: significant increases in family size compared to outgroups, an increase of three or more sequences (i.e. more than WGD would provide) in *P. antipodarum* compared to the other species, and at least one sequence in all five species analyzed for GO enrichment analysis, as described above.

## Supplementary Material

evaf192_Supplementary_Data

## Data Availability

All raw sequencing data are available at NCBI in the SRA (SRR29047464, SRR29047466, SRR29099380, SRR29099381, SRR29099382, SRR29099389, and SRR32688197) under BioProject PRJNA717745. The uncollapsed contig assembly is available at NCBI under accession JBMDTV000000000. The version described in this paper is version JBMDTV010000000. Both the uncollapsed and collapsed assemblies, transcriptome assemblies, all annotations, and gene trees are available on Zenodo (https://doi.org/10.5281/zenodo.15015201). All scripts developed for this project, as well as all supplemental information and flow cytometry data, are available on GitHub (https://github.com/jsharbrough/potamomics). Alex Yellow snails are available on request.
